# Pain Management in Childhood Leukemia: Diagnosis and Available Analgesic Treatments

**DOI:** 10.3390/cancers12123671

**Published:** 2020-12-07

**Authors:** Flaminia Coluzzi, Monica Rocco, Rula Green Gladden, Pietro Persiani, Laurel A. Thur, Filippo Milano

**Affiliations:** 1 Department Medical and Surgical Sciences and Biotechnologies, Sapienza University of Rome, Polo Pontino, 04100 Latina, Italy; 2 Unit Anesthesia, Intensive Care and Pain Medicine, Sant’Andrea University Hospital, 00189 Roma, Italy; monica.rocco@uniroma1.it; 3 Department of Surgical and Medical Science and Translational Medicine, Sapienza University of Rome, 00189 Roma, Italy; 4 Seattle Children’s Hospital, Seattle, WA 98105, USA; Rula.GreenGladden@seattlechildrens.org; 5 Department of Medicine, University of Washington School of Medicine, Seattle, WA 98195, USA; fmilano@fredhutch.org; 6 Department of Anatomical, Histological, Forensic Medicine and Orthopedic Science, Sapienza University of Rome, 00185 Rome, Italy; pietro.persiani@uniroma1.it; 7 Clinical Research Division, Fred Hutchinson Cancer Research Center, Seattle, WA 98109, USA; lthur@fredhutch.org

**Keywords:** leukemia, children, opioids, osteonecrosis, chronic pain, neuropathic pain, musculoskeletal pain

## Abstract

**Simple Summary:**

Leukemia, the most common form of childhood cancer, accounts for about 30 percent of all cancers that affect children and young adults. Although pain is a common and distressing symptom of childhood leukemia, evidence-based guidance regarding assessment and management is lacking. With 6 international multidisciplinary healthcare professionals, we aimed to review current evidence in literature, addressing diagnosis, assessment and pharmacological management of the most common leukemia-related pain syndromes in children, including cancer-related painful neuropathies and osteonecrosis. We also focus on the impact of pain on quality of life of childhood leukemia patients and the most common pain medications used in the pediatric population, with differences in their utilizations across countries.

**Abstract:**

Pain is one of the most common symptoms in children suffering from leukemia, who are often misdiagnosed with other childhood painful diseases such as juvenile idiopathic arthritis. Corticosteroid-induced osteonecrosis (ON) and vincristine-induced peripheral neuropathy (VIPN) are the most common painful manifestations. Additionally, ongoing pain may continue to impact quality of life in survivorship. This narrative review focuses on the pathophysiological mechanisms of pain in childhood leukemia and current available indications for analgesic treatments. Pain management in children is often inadequate because of difficulties in pain assessment, different indications across countries, and the lack of specific pediatric trials. Analgesic drugs are often prescribed off-label to children by extrapolating information from adult guidelines, with possible increased risk of adverse events. Optimal pain management should involve a multidisciplinary team to ensure assessment and interventions tailored to the individual patient.

## 1. Prevalence of Pain in Childhood Leukemia

Leukemia is the most frequent cancer among pediatric patients, accounting for 25% of childhood malignancies [1], where acute lymphoblastic leukemia (ALL) is the most common type with over three quarters of diagnoses occurring in developed countries. The highest rates of ALL are in children aged one to four years old [2]. About 40% of children with ALL suffer from bone and joint pain, which is the most common presenting symptom after fatigue and fever [3]. Unfortunately, over 75% of children with osteoarticular pain are misdiagnosed as suffering from juvenile idiopathic arthritis (JIA) [4]. Time from the first clinical manifestation and a bone marrow examination may extend to several months [5]. Differential diagnoses for osteoarticular pain and fever in children include lymphoma, osteomyelitis, primary bone or metastatic malignancies, septic arthritis, and autoimmune diseases^2^. At the time of manifestation of musculoskeletal symptoms, when most children are evaluated by rheumatologists, up to 75% do not have blasts in the peripheral blood [6]. Bone pain and fever, particularly if associated with fatigue and bruising, should lead to prompt consideration of ALL diagnosis. The aim of this review was to investigate the pathogenesis of pain in childhood leukemia and the currently available therapeutic options.

## 2. Pathogenesis of Pain in Childhood Leukemia

Musculoskeletal pain is quite common among children with ALL before diagnosis and during treatment. In the early stage of ALL, the most common musculoskeletal manifestations are arthralgia, arthritis, myalgia, and bone pain [4]. During the maintenance phase, pain may be the result of treatment complications such as corticosteroid-induced osteonecrosis (ON) and vincristine-induced peripheral neuropathy (VIPN).

Even though leukemia cells have not directly infiltrated the synovium, children may report arthralgia for the periosteal lesions of the juxta-articular bones [7]. According to the literature, 18% of ALL children suffer from arthropathy, with a similar distribution between arthritis (9%) and arthralgia (9%). Usually one or two joints are involved, with hip, knee, and ankle being the most common. In these patients, the percentage of misdiagnoses reaches 88% of cases [8].

## 3. Pain Assessment in Children

An accurate assessment of pain in ALL children is important for diagnosis and treatment; however, it is challenging, particularly in children younger than six years, because pain can be difficult to distinguish from other causes of discomfort. The new definition of “pain”, updated in July 2020 by the International Association for the Study of Pain (IASP), stressed the concept that “verbal description is only one of several behaviors to express pain; inability to communicate does not negate the possibility that a human or a nonhuman animal experiences pain” [9]. Specific tools have been validated for children unable to use the Numeric Rating Scale (NRS) or the Visual Analogue Scale (VAS), which represent the gold standard in adults and in children aged eight and above. The Oucher Pain Scale™, available in five different ethnic pictures, and the Wong–Baker Faces Scale, draw a spectrum of facial expressions, which are useful for pain assessment in younger children, but are not valid in those less than three years of age [10].

Diagnosing neuropathic pain is further challenging in children but is essential for the correct selection of the analgesic therapy and requires specific tools. The pediatric-modified Total Neuropathy Score (ped-mTNS) was introduced in 2009, consisting of an interview-based questionnaire and physical examination [11]. This tool has been validated in different languages, recently in a Dutch pediatric oncology population that included children with ALL [12]. The ped-mTNS contains eight questions about sensory, functional, and autonomic symptoms. Physical examination assesses five specific aspects: light-touch sensation, pin sensibility, muscle strength, and deep tendon reflexes of the Achilles and patella. For all items in the questionnaire and the physical examination part, the score ranged between 0 (no symptoms) and 4 (severe symptoms), with a total score ranging from 0 to 32. Children with scores ≥5 are considered affected by VIPN. Some limitations remain in younger patients. No pediatric neuropathic pain tools have been validated for children younger than five years.

## 4. Indications and Limits of Currently Available Analgesics for Children

Pain management requires adequate pain assessment in terms of pain intensity and pain quality. With regard to pain intensity, the World Health Organization (WHO) analgesic ladder has been shown to be an effective instrument for managing children with leukemia [13]. Among ALL patients with arthropathy, paracetamol and non-steroid anti-inflammatory drugs (NSAIDs) are the most commonly prescribed drugs in about 40–65% of patients [4,8]. Paracetamol (acetaminophen) is the most commonly prescribed analgesic in children, as a monotherapy or as an add-on to established treatment with strong opioids. However, there is no high-quality evidence to support or discourage the use of paracetamol in pediatric cancer pain [13]. In ALL children, paracetamol can also be used as a premedication, together with methylprednisolone and diphenhydramine, for the prevention of allergic reactions induced by transfusions and PEG-L-asparaginase (pegaspargase) [14]. Intra-articular corticosteroids are prescribed in about 20% of children with ALL-related arthritis because they are initially misdiagnosed as JIA [8]; however, the use of corticosteroids may further delay the diagnosis.

In moderate-to-severe pain, opioids continue to represent a cornerstone of therapy, however, insufficient evidence exists on the safety and efficacy of opioids in the pediatric population. Approved drugs differ across countries and most opioids are currently prescribed off-label in the pediatric setting. Weak opioids such as codeine and tramadol have been commonly prescribed in the pediatric population for pain medicine [15]. They are available with or without paracetamol, are quite inexpensive, available in different formulations, and considered well tolerated. However, recent concerns about their safety have completely changed their role in pain management in children.

Prescription of codeine and tramadol has been restricted in the U.S. by the Food and Drug Administration (FDA) in children younger than 12 years old because of numerous adverse events and fatalities [16]. In addition to these restrictions, there is a warning against their use in adolescents between 12 and 18 years who are obese or suffering from obstructive sleep apnea (OSAS) or severe lung disease due to the increased risk of serious breathing problems [17]. The major concern for their restriction was, indeed, the potential to cause harmful and even fatal respiratory depression due to variable cytochrome expression and altered metabolism in ultra-rapid metabolizers (UM) patients [18].

In Europe, the European Medicines Agency’s (EMA) Pharmacovigilance Risk Assessment Committee (PRAC) has recommended restrictions on the use of codeine-containing medicines in children below 12 years because of the risk of serious side effects including breathing problems [19]. Tramadol is currently approved by EMA in children older than one year and is mainly used for acute nociceptive pain management. In Europe, its use has been recently restricted for postoperative pain management in children with certain conditions such as OSAS and compromised respiratory function [20]. In 2017, in Australia, where weak opioids are not recommended for children younger than 12 years, they accounted for 60.7% of the prescribed opioids, with codeine being the most commonly dispensed opioid (50.5% of dispensed prescriptions) [21].

According to the Pediatric Health Information System (PHIS) database, from over 40 tertiary care pediatric hospitals in the U.S., 95.2% of children with acute myeloid leukemia (AML) received analgesic treatment, and 77.7% were exposed to opioids. Morphine, fentanyl, and oxycodone were the most prescribed opioids, with a rate of 47.9%, 38.2%, and 22.6%, respectively. Intravenous opioids were more commonly prescribed than oral formulations (68.8 vs. 44.5%). The likelihood of opioid exposure varied consistently by age (children higher than 10 years were more likely to receive opioids), while no differences in prevalence were observed by gender, race, or insurance [22]. Low dose strong opioids are becoming the alternative to weak opioids. Morphine remains the most common alternative to codeine in pediatric patients because it is available in different formulations, multiple strengths, and allows flexible dosing regimens. When using opioids, neonates are at higher risk for central respiratory depression because of immaturity of hepatic enzymes, renal function, and hypoxic respiratory drive centers [23].

Oxycodone was approved in 2016 by the FDA for children between 11 and 16 years old, when other analgesic drugs did not provide adequate pain management. In Europe, oxycodone is not recommended for children younger than 12 years, given the lack of clinical data on its use in this setting. Oxycodone is unlikely to be a safer alternative than codeine or tramadol in children considering that the concentration of its active metabolite, oxymorphone may increase in UM [24]. Another alternative is the hydrocodone/acetaminophen combination, which is currently indicated for moderate to severe pain in children aged two years and over. Pharmacokinetics (PK) of this association in children resembles that observed in adults [25].

Tapentadol is an innovative atypical opioid with a dual mechanism of action, mu-opioid receptor (MOR) agonism, and noradrenaline reuptake inhibition. Tapentadol has been widely used in different cancer pain populations including patients with pain from hematological malignancies [26] and patients experiencing chemotherapy-induced neuropathic pain conditions [27]. Due to its lower μ-opioid load (MOR affinity is 50 times lower than that of morphine), tapentadol has a favorable tolerability profile, in terms of gastrointestinal adverse events [28,29] and endocrine effects such as no significant changes in testosterone concentration [30]. Furthermore, this molecule is associated with a low risk of drug–drug interactions at the CYP450 level, and may therefore be used in poly-treated patients [31]. Tapentadol is the first and only drug in Europe to have been developed by a multinational pediatric drug development program for children aged 2 to <8 years of age [32]. Tapentadol is available for children as an oral solution, for the treatment of moderate to severe acute postoperative pain [33,34,35], with adequate palatability and acceptability to ensure intake compliance in all age groups. To date, tapentadol has not been approved for children in the U.S.

Regarding transdermal (TTS) formulations, TTS-fentanyl has been used in children with cancer pain including AML when the oral route of administration was not tolerated or its elimination was judged likely to improve the quality of life (QoL) [36]. The few published reports about fatal TTS-fentanyl overdoses have been caused by accidental patch application by healthy children, and point to the need for adequate education of users on handling of this potent analgesic [37,38]. Initial evidence for feasibility and tolerability of fentanyl-based rapid onset formulations for breakthrough pain in children have been published such as lozenge [39] and intranasal administration [40].

Few reports are available on the use of TTS-buprenorphine in children with cancer pain [41]. In an open-label non-randomized trial, TTS-buprenorphine, administered following a 72-h schedule, resulted in being efficient, safe, and well tolerated in children with moderate to severe cancer related pain [42]. Among strong opioids, case studies suggest a potential role for methadone in the treatment of cancer-related pain in children with indications for nociceptive, neuropathic, and mixed syndromes [43]. Side effects are mild and mainly include nausea, vomiting, confusion, and sedation. No clinically relevant arrhythmias have been detected in children. The minimum age for the use of common pediatric analgesics based on EMA and FDA approval is described in Table 1.

## 5. Treatment of Pediatric Cancer-Related Neuropathic Pain

Long-term small-fiber neuropathy (SFN) and altered pain sensitization have been observed in relapse-free long-term survivors of pediatric ALL after stem cell transplantation [44]. Quantitative Sensory Testing (QST) was used to test almost all somatosensory aspects as it is a reliable assessment to rule out SFN [45]. A total of 96% of ALL survivors showed at least one abnormal parameter in QST, the most common being increased thresholds for vibration (56%), tactile detection (40%), and decreased thresholds for mechanical nociceptive stimulation (32%).

One of the main causes of neuropathic pain in children with leukemias is because of VIPN. The neurotoxicity of vincristine induces a mixed sensory, motor, and autonomic neuropathy, with longer peripheral nerves being more susceptible. Peripheral neuropathy is typically symmetrical, distal, and progresses proximally. Children complain of paresthesia, numbness, tingling, loss of proprioception, and pain. Autonomous nervous system involvement causes constipation and dizziness. Multiple risk factors have been associated with VIPN including administered dose, age (older are more susceptible than younger), race (white race is at an increased risk over non-white), and genetic factors, related to the CYP3A4/5 pathway [46]. Similarly, nelarabine has been reported to induce dose-dependent neurotoxicity [47]. However, these drugs remain the most important medications for the curative treatment of ALL, and there are no clear guidelines around holding. As most of the drug-induced neuropathies are ultimately reversible, most studies recommend their use at full dose.

Most of the literature on the treatment of pediatric cancer-related neuropathic pain (NP) is related to chemotherapy-related syndromes, followed by tumor-related and post-surgical NP [48]. In NP syndromes, adjuvants such as anticonvulsant or antidepressants are recommended as first-choice drugs. Despite the extensive data supporting their use in adults with NP, evidence on the clinical use of gabapentinoids as treatment options for pain in children and adolescents is still fair [49,50].

Gabapentin is probably the most used analgesic in children suffering from VIPN. A retrospective analysis of 498 ALL patients showed that 62.2% of children suffering from NP were treated with gabapentin, but did not conclusively demonstrate its efficacy [48]. In a recent prospective randomized, double-blind, placebo-controlled trial conducted in children with VIPN, the opioid consumption was higher in patients treated with gabapentin compared with the placebo [51]. The main limitations of this trial were the lack of dose escalation, which is typical of gabapentin’s use in pain management, and the limited three week duration.

In an open-label trial conducted on children (aged 10–17 years) treated with chemotherapy for solid tumors and leukemia, pregabalin was shown to be effective in reducing the pain of chemotherapy-induced neuropathy. Compared with gabapentin, pregabalin allows a faster titration with a good tolerability profile and 93% of patients completed the follow up [52]. However, its use in children suffering from NP is off-label. Pregabalin has recently been approved as adjunctive therapy for the treatment of partial onset seizures in patients four years of age and older. While it is not authorized for use in Europe, a Pediatric Post Marketing Pharmacovigilance Review by the FDA in 2019 did not report any pediatric safety concerns for pregabalin.

Contrary to adults, there are not enough data on the use of antidepressants to support their use to treat NP in children and adolescents [50]. A randomized clinical trial in NP in children showed no difference between amitriptyline and gabapentin in terms of pain reduction, sleep quality, and tolerability [53]. Prior to starting a therapy with tricyclic antidepressants (TCAs), an electrocardiogram is recommended [54]. Lower dosages are mandatory in children with significant slowing of cardiac conduction. Among the antidepressants, selective noradrenaline and serotonin inhibitors (SNRIs) are easier to use in clinical practice because of the lack of anticholinergic and antihistaminergic effects, typical of TCAs, but no data exist on pediatric NP management.

## 6. Osteonecrosis in Childhood Leukemia: Treatment Strategies and Role of Biphosphonates (BPs)

One particularly debilitating cause of pain in survivors of pediatric ALL is ON. The incidence of symptomatic ON at the end of leukemia treatment ranges from 0.9% [55] to 17.6% [56]. After one year of therapy, approximately 15% of patients with ALL displayed radiological evidence of ON using magnetic resonance imaging (MRI) [57]. Adolescents are more susceptible than younger children to develop ON [58]. Pain is often the first symptom leading to the diagnosis of ON [59], and its severity is strictly related to the joint functionality and can involve multiple joints [58,60]. However, there are ALL children with MRI evidence of ON who do not report joint pain at all, or only occasional pain [57].

ON in pediatric ALL patients is strongly associated with glucocorticoid use, but the exact pathophysiology by which glucocorticoids cause ON is not well understood. Roles have been suggested for altered lipid metabolism, thrombophilia, and bone development [56,61]. In pediatric patients with ALL, corticosteroids are another therapeutic cornerstone. The general consensus is to not modify steroid therapy for ON during induction or delayed intensification, while it is possible to omit maintenance therapy in the case of ON Grade 2 or greater. In the latter circumstance, it is possible to consider resuming maintenance with steroids after six months, if joint symptoms have resolved and if MRI findings have significantly improved or normalized [62].

In addition to steroids, radiation increases risk for ON; there is a higher incidence of ON in hematopoietic stem cell transplant (HSCT) survivors who received total body irradiation (TBI), likely secondary to radiation-induced microvascular damage. Graft versus host disease (GvHD) further increases risks via associated microangiopathy and prolonged exposure to glucocorticoid [63,64].

There is no consensus regarding screening or treatment algorithms for ON. However, current treatment options for ON include analgesic medications in combination with limited weight bearing, physical therapy, and surgery. Key surgical procedures include core compression, joint replacement, vascularized bone grafts, or core decompression with insertion of human morphogenetic protein. There is no single agreed-upon surgical approach in pediatric ON, in part due to the young age and skeletal immaturity of pediatric patients. In general, total joint replacement is delayed as long as possible as children are expected to significantly outlive their prostheses [63,65]. Promising new approaches include core decompression with implantation of autologous mesenchymal stem cell therapy [66] and the use of hyperbaric oxygen therapy (HBOT) [67,68]. Controversial is the use of medical therapies targeting pathophysiologic mechanisms such as bone metabolism, lipid metabolism or microvascular health, and including calcium channel blockers, prostaglandins (iloprost), low molecular weight heparin, statins, and bisphosphonates [63,65]. Similarly, there have been no large studies of bisphosphonates for ON in childhood leukemia survivors and for this reason, indication and expected outcomes for treatment remain unclear [69,70,71,72,73].

## 7. The Experience of Pain and Impact on Quality of Life

The acute and chronic pain experienced during treatment impacts the QOL of childhood leukemia patients during therapy. Duipis et al. assessed children’s QOL using indexes including pain subscales over the first year of therapy for standard risk ALL. Pain was given a score using these indexes rather than being reported as present or not present. Children experienced the most pain the month after diagnosis [74]. Similarly, prospective assessment of health-related quality of life (HRQoL) in leukemia patients undergoing treatment showed decreased HRQoL and elevated “pain and hurt” subscale scores, especially in the first month of treatment [75]. Additionally, in a prospective, international study of 118 children undergoing cancer therapy, 48% reported pain; patients with leukemia or lymphoma who reported pain had significantly lower HRQoL [76].

One method of analysis evaluates symptoms that occur in a cluster with other symptoms. During treatment, pain often occurs with other symptoms including fatigue, sleep disturbance, nausea, and depression. Analyzed as a cluster and in association with physical activity and cognition, children undergoing treatment for leukemia who had more severe symptoms had lower cognition, and when symptom severity worsened, so did their cognition. This suggests that pain in combination with other symptoms in the cluster may impact physical activity as well as cognition, and by extension, childhood development throughout treatment [77].

Following completion of treatment, pain continues to impact the quality of life of childhood leukemia survivors. As high as 41% of childhood leukemia survivors experience pain [78]. Meekse et al. found that 30% of childhood leukemia survivors reported pain, and survivors who experienced chronic fatigue were five times more likely to report pain than non-fatigued survivors [79]. Pain was additionally independently and significantly associated with depression, while fatigue and depression were inversely related to QOL [79]. A more recent study evaluating chronic fatigue in childhood leukemia and lymphoma survivors in Norway found that survivors with persistent chronic fatigue were more likely to report pain and its interference in daily activities [80]. They found that leukemia and lymphoma survivors with chronic fatigue were more likely to have muscle or joint pain and share some but not all biomarkers seen in chronic fatigue syndrome [81].

Pain may impact overall quality of life assessment. Childhood AML survivors who underwent chemotherapy with or without autologous or allogenic HSCT reported HRQoL scores similar to those of the general population, whereas survivors who reported more chronic health conditions and cancer-related pain had lower QOL [78]. Similarly, researchers in Canada found that long-term survivors of pediatric ALL had overall good HRQoL, but there was a specific group of lower HRQoL with disabilities in the areas of hearing, emotion, cognition, and pain [82]. Child survivors with a history of ON severe enough to require surgical intervention reported severely impaired functional outcomes and poor HRQoL [83].

There are several informative reports from the Childhood Cancer Survivor Study (CCSS), which followed more than 9000 patients with a variety of pediatric cancer diagnoses from 1970 to 1986 including survivors of childhood leukemia. One report surveyed patients on average from 17 years from diagnosis and found 8.3% of leukemia survivors had lower reports of pain “from cancer or its treatment” compared to other tumor types. While they used siblings as controls for other metrics, they did not query the siblings about pain, and therefore lacked a comparison group [84]. Survivors were more likely than their siblings to experience all pain metrics. However, there were no statistically significant differences in these metrics when analyzed by disease type. Interestingly, younger age at diagnosis was associated with all pain types, but was not associated with the attribution of pain to cancer treatment. Across cancer types, female gender, minority status, lower socioeconomic status, unemployment, and being single were significantly associated with pain conditions [85]. An additional report from the CCSS evaluating ongoing emotional distress found that survivors of leukemia were more likely to experience emotional distress than other cancer types and this was associated with bodily pain [86]. Finally, another study from the CCSS found that while improvements in treatment and supportive care have improved survival in childhood leukemia, self-reported poor general health, cancer-related pain, and anxiety have increased over time [87].

Some studies have evaluated specific types of pain. Researchers in Texas evaluated prevalence of back pain in survivors of pediatric ALL at least five years out from end of treatment and found increased prevalence of back pain in survivors, with 44% of survivors reporting back pain relative to 22% of their siblings [88]. Similarly, researchers at St. Jude Children’s Hospital evaluated headache in ALL survivors and found that while headache prevalence was similar to the prevalence in the general population (in published literature; they lacked a control group), there was increased prevalence of particular headache types: migraine headaches (31% versus 7–9% in the general population), tension-type headaches (30% versus 10–25%), and chronic headaches (11% versus 3.5%) [89]. Decreased mental and emotional quality of life was associated with migraine and tension-type headaches. Although they lacked a control group, at least one neurologic symptom was present in 83% of long-term ALL survivors. While they found that symptoms-related morbidity and QOL impairment was low in the majority of survivors, female sex, ≥10 doses of intrathecal chemotherapy, and history of ALL relapse were associated with decreased QOL [90].

## 8. Conclusions

Pain is a common feature in childhood leukemia, often misdiagnosed and not adequately treated. Many factors may lead to difficulties in diagnosing different types of pain and selecting effective treatment strategies. Most analgesic drugs used in pediatric care have not been licensed for children or for a specific type of pain, particularly in neuropathic pain conditions. However, off-label use is often unavoidable because of the lack of labeled therapies, but this increases the risk of adverse events and lack of efficacy. Specific pediatric trial programs are warranted for new analgesics. Optimal pain management should involve a multidisciplinary team to ensure assessment and interventions tailored to the individual patient. Thoughtful pain management continues to be required in survivorship, when ongoing pain may continue to impact quality of life.

## Figures and Tables

**Table 1:** Minimum age for common pediatric analgesics.

Drug	EMA (Europe)	FDA (U.S.)
**NSAIDs/APAP**		
Diclofenac	6 yrs	Not authorized
Ibuprofen	3 mo	6 mo.
Ketoprofen	>20 kg	Not authorized
Naproxen	1 yr and >10 kg	2 yrs (rheumatoid arthritis)
Metamizole	4 mo. ^a^	Not authorized (removed from U.S. market in 1977)
Paracetamol (acetaminophen)	Neonates	Neonates
**Opioids**		
Codeine/acetaminophen	12 yrs	12 yrs
Tramadol	1 yr and >10 kg	12 yrs
Morphine	Neonates	Not authorized
Oxycodone	12 yrs	11 yrs (extended release)
Tapentadol	2 yrs	Not authorized
Methadone	Not authorized	Not authorized
TTS Fentanyl	2 yrs	2 yrs
TTS Buprenorphine	18 yrs	16 yrs (subdermal implant for opioid dependence)
**Anticonvulsants**		
Gabapentin	6 yrs	3 yrs ^b^
Pregabalin	Not authorized	1 mo (as adjunctive agent for treatment of partial seizure)
**Antidepressants**		
Duloxetine	Not authorized ^c^	7 yrs or 13 yrs (7 yrs for generalized anxiety disorder, 13 yrs for fibromyalgia)
Venlafaxine	Not authorized ^c^	Not authorized
Amitriptyline	Not authorized	12yrs (depression)

^a^ Do not use the IV formulation in neonates aged 3–11 months. Drops are indicated for neonates ≥ 4 months, suppositories for children ≥ 4 years, and pills for children ≥ 15 years. ^b^ As adjunctive therapy in the treatment of partial seizures in pediatric patients ≥ 3 years. Off-label use for neuropathic pain in adults. No data available in the management of neuropathic pain in pediatric patients. ^c^ In the case of off-label use in children, the appearance of suicide-related behaviors should be carefully monitored. Abbreviations: EMA: European Medicines Agency; FDA: U.S. Food and Drug Administration; iv: intravenous; kg: kilogram; mo: months; NSAIDs/APAP: Non-Steroidal Anti-Inflammatory Drug/Acetaminophen; TTS: Transdermal Therapeutic System; U.S.: United States; yrs: years.

## Precis for Use in the Table of Contents

Pain management in children is often inadequate because of difficulties in pain assessment, different indications across countries, and the lack of specific pediatric trials. Most analgesic drugs used in pediatric care have not been licensed for children or for a specific type of pain, particularly in neuropathic pain conditions.
